# The obstacle problem for conformal metrics on compact Riemannian manifolds

**DOI:** 10.1186/s13660-018-1827-3

**Published:** 2018-09-12

**Authors:** Sijia Bao, Yuming Xing

**Affiliations:** Department of Mathematics, Harbin Institute of Technology, Harbin, China

**Keywords:** Obstacle problem, A priori estimates, Hessian equations, Viscosity solutions, Riemannian manifolds

## Abstract

We prove a priori estimates up to their second order derivatives for solutions to the obstacle problem of curvature equations on Riemannian manifolds $(M^{n}, g)$ arising from conformal deformation. With the a priori estimates the existence of a $C^{1,1} $ solution to the obstacle problem with Dirichlet boundary value is obtained by approximation.

## Introduction

Let $(M^{n}, g)$ be a compact Riemannian manifold of dimension $n \geq3$ with smooth boundary *∂M*, $\bar{M}:= M \cup \partial M$. In conformal geometry, it is interesting to find a complete metric $\tilde{g} \in[g]$, the conformal class of *g*, with which the manifold has prescribed curvature. In general, such conformal deformation can be interpreted by certain partial differential equations. See [8, 13, 22, 25, 26] for more details.

In [8], Guan studied the existence of a complete conformal metric *g̃* of negative Ricci curvature on *M* satisfying

1.1 $$ f \bigl( - \lambda\bigl(\tilde{g}^{-1} \mathrm{Ric}_{\tilde{g}}\bigr)\bigr) = \psi\quad \mbox{in } M, $$

where $\mathrm{Ric}_{\tilde{g}}$ is the Ricci tensor of *g̃*, and $\lambda(\tilde{g}^{-1} \mathrm{Ric}_{\tilde{g}}) = (\lambda_{1}, \ldots , \lambda_{n})$ are the eigenvalues of $\tilde{g}^{-1} \mathrm {Ric}_{\tilde{g}}$. The transformation formula for the Ricci tensor under conformal deformation $\tilde{g} = e^{2 u} g$ is given by

$$\frac{1}{n - 2} \mathrm{Ric}_{\tilde{g}} = \frac{1}{n - 2} \mathrm{Ric}_{g} - \nabla^{2} u - \biggl(\frac {\Delta u}{n - 2} + \vert \nabla u \vert ^{2} \biggr) g + du \otimes du, $$

where ∇*u*, $\nabla^{2} u$, and Δ*u* denote the gradient, Hessian, and Laplacian of *u* with respect to the metric *g*, respectively. When *f* is homogenous of degree one, it is easy to verify that equation (1.1) is equivalent to the following form:

1.2 $$ f \biggl(\lambda \biggl(g^{-1} \biggl[ \nabla^{2} u + \frac{\Delta u}{n - 2} g + \vert \nabla u \vert ^{2} g - du \otimes du - \frac{\mathrm{Ric}_{g}}{n - 2} \biggr] \biggr) \biggr) = \frac{\psi(x)}{n - 2} e^{2u}. $$

In this paper, we study the obstacle problem of equation (1.2). More generally, let

$$T [u] := \nabla^{2} u + s \, du \otimes du + \biggl( \gamma\Delta u - \frac{t}{2} \vert \nabla u \vert ^{2} \biggr) g + \chi, $$

where *χ* is a smooth $(0,2)$ tensor, $\gamma> 0$ is a constant, and $s, t \in\mathbb{R}$. We consider the following equation:

1.3 $$ \max \bigl\{ u -h, - \bigl(f \bigl(\lambda\bigl(g^{-1} T [u]\bigr)\bigr) - \psi[u]\bigr) \bigr\} = 0 \quad\mbox{in } M $$

with the Dirichlet boundary condition

1.4 $$ u = \varphi\quad\mbox{on } \partial M, $$

where $h \in C^{3} (\bar{M})$, $\varphi\in C^{4} (\partial M)$, $h > \varphi$ on *∂M*, $\psi[u] = \psi(x, u)$ is a positive function in $C^{3} (\bar{M} \times\mathbb{R})$.

Equations as (1.1) and (1.3) are the Hessian equations, which were well studied by many authors such as [2, 7, 9–12, 23, 24]. Generally, $f \in C^{2} (\Gamma) \cap C^{0} (\bar{\Gamma})$ is a symmetric function of $\lambda\in\mathbb{R}^{n}$, defined in an open, convex, and symmetric cone $\Gamma\subsetneqq\mathbb{R}^{n}$, with vertex at the origin, which contains the positive cone: $\Gamma_{n}^{+} : =\{\lambda\in\mathbb{R}^{n}: \mbox{each component } \lambda_{i}>0\}$ and satisfies the following fundamental structure conditions:

1.5 $$ f_{i} \equiv\frac{\partial f}{\partial\lambda_{i}} > 0\quad \mbox{in } \Gamma, 1 \leq i \leq n, $$

1.6 $$ \mbox{$f$ is a concave function}, $$

and

1.7 $$ f > 0 \quad\mbox{in } \Gamma, \qquad f = 0 \quad\mbox{on } \partial\Gamma. $$

Here, for convenience, we also assume that

1.8 $$ \mbox{$f$ is homogeneous of degree one}. $$

We observe that by the concavity and homogeneity of *f*,

1.9 $$ \sum f_{i} (\lambda) = f (\lambda) + \sum f_{i} (\lambda) (1 - \lambda _{i}) \geq f (1, \ldots, 1) > 0 \quad\mbox{in } \Gamma. $$

Important classes of *f* are the elementary symmetric functions and their quotients, i.e.,

$$f (\lambda) = (\sigma_{k})^{\frac{1}{k}} (\lambda) : = \biggl(\sum _{1 \leq i_{1} < \cdots< i_{k} \leq n} \lambda_{i_{1}} \cdots \lambda_{i_{k}} \biggr)^{\frac {1}{k}}, \quad 1 \leq k \leq n, $$

and

$$f (\lambda) = \biggl(\frac{\sigma_{k}}{\sigma_{l}} \biggr)^{\frac {1}{k-l}}, \quad0 \leq l < k \leq n. $$

Let *F* be defined by $F (r) = f (\lambda(r))$ for $r = \{r_{ij}\} \in\mathcal{S}^{n \times n}$ with $\lambda(r) \in\Gamma$, where $\mathcal{S}^{n \times n}$ is the set of $n \times n$ symmetric matrices. It is shown in [2] that (1.5) implies *F* is an elliptic operator and (1.6) ensures that *F* is concave.

A function $u \in C^{2} (M)$ is called *admissible* at $x \in M$ if $\lambda(g^{-1} T [u]) (x) \in\Gamma$, and we call it *admissible* in *M* when it is admissible at each *x* in *M*. In this paper, we prove the existence of an admissible viscosity solution of (1.3) and (1.4) in $C^{1, 1} (\bar{M})$ (see [1, 3] for the definition of viscosity solutions).

Many authors have studied various obstacle problems. In [6], Gerhardt considered a hypersurface bounded from below by an obstacle with prescribed mean curvature in $\mathbb{R}^{n}$. Lee [17] considered the obstacle problem for the Monge–Ampère equation (i.e., $f = (\sigma_{n})^{\frac{1}{n}}$) for the case that $T [u] = D^{2} u$, $\psi\equiv1$, and $\varphi\equiv0$, and proved the $C^{1,1}$ regularity of the viscosity solution in a strictly convex domain in $\mathbb{R}^{n}$. Xiong and Bao [27] extended the work of Lee to a nonconvex domain in $\mathbb{R}^{n}$ with general *ψ* and *φ* under additional assumptions. Bao, Dong, and Jiao treated a class of obstacle problems in [1] assuming that $T[u] = \nabla^{2} u + A(x, u, \nabla u)$, under a certain technical assumption. Because of the term $\gamma\Delta u$ ($\gamma> 0$), here we only need a minimal amount of assumptions. For other works, see [4, 14, 15, 18–21].

Our main result is the following theorem.

### Theorem 1.1

*Assume that* (1.5)*–*(1.8) *and either the following condition*

1.10 $$ \lim_{z \rightarrow+ \infty} \psi(x, z) = +\infty, \quad\forall x \in\bar{M}, $$

*or*

1.11 $$ \frac{2s - n t}{1 + n\gamma} < 2 \lambda_{1} $$

*hold*, *where* $\lambda_{1}$ *is the first eigenvalue of the problem*

1.12 $$ \textstyle\begin{cases} \Delta u + \lambda(\operatorname{tr} \chi)^{+} u = 0 & \textit{on } \bar{M},\\ u = 0 & \textit{on } \partial M \end{cases} $$

($\lambda_{1} = + \infty$ *if* $\operatorname{tr} \chi\leq0$). *Then there exists a viscosity solution* $u \in C^{1,1} (\bar{M})$ *to* (1.3) *and* (1.4), *if there exists a subsolution* $\underline{u} \in C^{0} (\bar{M}) \cap C^{1} (\bar{M} _{\delta})$ *for some* $\delta> 0$ *such that*

1.13 $$ \textstyle\begin{cases} f (\lambda(g^{-1} T [\underline{u}])) \geq\psi[\underline{u}], & \textit{in } M,\\ \underline{u} = \varphi, & \textit{on } \partial M, \\ \underline{u} \leq h, & \textit{in } M, \end{cases} $$

*where* $M_{\delta}= \{x \in M: \operatorname{dist} (x, \partial M) \leq \delta\}$. *Moreover*, *we have that* $u \in C^{3, \alpha} (E)$ *for any* $\alpha\in (0, 1)$, *and* $f(\lambda(g^{-1} T [u])) = \psi[u]$ *in* *E*, *where* $E:= \{x \in M: u (x) < h (x)\}$.

### Remark 1.2

(1.10), as well as (1.11), is used in Lemma 3.2 to derive an upper bound for *u*. Assumption (1.13) is just applied to derive a lower bound for *u* on *M* and $\nabla_{\nu} u$ on *∂M*, where *ν* is the interior unit normal to *∂M*.

### Remark 1.3

We can construct some subsolutions of (1.2) satisfying (1.13) as in [15] following ideas from [2] and [7] since

$$\vert \nabla u \vert ^{2} g - du \otimes du $$

is positive definite and that we can obtain a priori upper bound of any admissible function (Lemma 3.2) under additional conditions that there exists a sufficiently large number $R > 0$ such that at each point $x \in\partial M$,

1.14 $$ (\kappa_{1}, \ldots, \kappa_{n-1}, R) \in \Gamma, $$

where $\kappa_{1}, \ldots, \kappa_{n-1}$ are the principal curvatures of *∂M* with respect to the interior normal, and that for every $C > 0$ and every compact set *K* in Γ there is a number $R = R (C, K)$ such that

1.15 $$ f (R \lambda) \geq C \quad\mbox{for all } \lambda\in K. $$

We use a penalization technique to prove the existence of viscosity solutions to (1.3) and (1.4). We shall consider the following singular perturbation problem:

1.16 $$ \textstyle\begin{cases} f (\lambda( g^{-1} T [u] )) = \psi[u] + \beta_{\varepsilon}(u-h) & \mbox{in } M,\\ u = \varphi& \mbox{on } \partial M, \end{cases} $$

where the penalty function $\beta_{\varepsilon}\in C^{2} (\mathbb{R})$ satisfies

1.17 $$ \begin{aligned} & \beta_{\varepsilon}, \beta'_{\varepsilon}, \beta''_{\varepsilon}\geq 0 \quad\mbox{on } \mathbb{R}, \beta_{\varepsilon}(z) = 0, \mbox{ whenever } z \leq0; \\ & \beta_{\varepsilon}(z) \rightarrow\infty\quad\mbox{as } \varepsilon \rightarrow0^{+}, \mbox{ whenever } z > 0. \end{aligned} $$

An example given in [27] is

1.18 $$ \beta_{\varepsilon}(z) = \textstyle\begin{cases} 0, & z \leq0,\\ z^{3} / \varepsilon, & z > 0, \end{cases} $$

for $\varepsilon\in(0, 1)$. Observe that $\underline{u}$ is also a subsolution to (1.16).

Let

$$\mathscr{U} = \bigl\{ u_{\varepsilon} | u_{\varepsilon} \in C^{4}( \bar{M}) \mbox{ is an admissible solution of } (1.16) \mbox{ with } u_{\varepsilon} \geq\underline{u} \mbox{ on } \bar{M} \bigr\} . $$

We aim to derive the uniform bound

1.19 $$ \vert u_{\varepsilon} \vert _{C^{2} (\bar{M})} \leq C $$

for $u_{\varepsilon}\in\mathscr{U}$, where *C* is independent of *ε*. After establishing (1.19), the equation (1.16) becomes uniformly elliptic by (1.7). By Evans–Krylov [5], [16] theorem, we can derive the $C^{2, \alpha}$ estimates (which may depend on *ε*) of $u_{\varepsilon}$. Higher estimates can be derived by Schauder theory. Following the proof as in [8] or [1], we can prove there exists an admissible solution $u_{\varepsilon}$ to (1.16). Then we can conclude by (1.19) that there exists a viscosity solution $u \in C^{1, 1} (\bar{M})$ to (1.3) and (1.4), see [1, 27].

Thus, our main work is focused on the a priori estimates for admissible solutions up to their second order derivatives. In Sect. 2, we achieve the estimates for second order derivatives. Finally, we end this paper with gradient and $C^{0}$ estimates in Sect. 3.

## Estimates for second order derivatives

In this section, we prove a priori estimates of second order derivatives for admissible solutions. From now on, we drop the subscript *ε* when there is no possible confusion.

### Theorem 2.1

*Assume that* *f* *satisfies* (1.5)*–*(1.8) *and* $u \in C^{4} (\bar{M})$ *is an admissible solution to* (1.16). *Then*

2.1 $$ \sup_{M} \bigl\vert \nabla^{2} u \bigr\vert \leq C \Bigl(1 + \sup_{\partial M} \bigl\vert \nabla^{2} u \bigr\vert \Bigr), $$

*where* *C* *depends on* $|u|_{C^{1}(\bar{M})}$ *and other known data*.

### Proof

Set

$$W(x) = \max_{ \xi\in T_{x} M, \vert \xi \vert = 1} \bigl( \nabla_{\xi\xi} u + s \vert \nabla_{\xi} u \vert ^{2} \bigr) e^{\phi}, \quad x \in\bar{M}, $$

where *ϕ* is a function to be determined. Assume that *W* is achieved at an interior point $x_{0} \in M$ and a unit direction $\xi\in T_{x_{0}} M$. Choose a smooth orthonormal local frame $e_{1}, \ldots, e_{n}$ about $x_{0}$ such that $\xi= e_{1}$, $\nabla_{i} e_{j} (x_{0}) = 0$ and that $T_{ij} (x_{0})$ is diagonal. We write $G = \nabla_{11} u + s |\nabla _{1} u|^{2} $. Assume $G (x_{0}) > 0$ (otherwise we are done).

At the point $x_{0}$, where the function $\log G + \phi$ (defined near $x_{0}$) attains its maximum, we have

2.2 $$ \frac{\nabla_{i} G}{G} + \nabla_{i} \phi= 0, \quad i = 1, \ldots, n, $$

and

2.3 $$ \frac{\nabla_{ii} G}{G} - \biggl(\frac{\nabla_{i} G}{G} \biggr)^{2} + \nabla_{ii} \phi\leq0. $$

By (2.3) we have

2.4 $$ F^{ii} \bigl(\nabla_{ii} G + G \nabla_{ii} \phi- G \vert \nabla_{i} \phi \vert ^{2} \bigr) \leq0 $$

and

2.5 $$ \Delta G + G \Delta\phi- G \vert \nabla\phi \vert ^{2} \leq0. $$

Since $\gamma> 0$, we obtain

2.6 $$ F^{ii} \bigl(\nabla_{ii} G + \gamma\Delta G + G \nabla_{ii} \phi+ \gamma G \Delta\phi- G \vert \nabla_{i} \phi \vert ^{2} - \gamma G \vert \nabla\phi \vert ^{2} \bigr) \leq0. $$

By calculation, we get

2.7 $$ \nabla_{i} G = \nabla_{i11} u + 2 s \nabla_{1} u \nabla_{i1} u, $$

and

2.8 $$ \nabla_{ii} G = \nabla_{ii11} u + 2 s \bigl( \vert \nabla_{i1} u \vert ^{2} + \nabla_{1} u \nabla_{ii1} u\bigr). $$

Recall the formula for interchanging order of covariant derivatives

2.9 $$ \nabla_{ijk} v - \nabla_{kij} v = R^{l}_{kij} \nabla_{l} v, $$

and

2.10 $$ \begin{aligned}[b] \nabla_{ijkl} v - \nabla_{klij} v = {}&R^{m}_{ljk} \nabla_{im} v + \nabla_{i} R^{m}_{ljk} \nabla_{m} v + R^{m}_{lik} \nabla_{jm} v \\ &{}+ R^{m}_{jik} \nabla_{lm} v + R^{m}_{jil} \nabla_{km} v + \nabla_{k} R^{m}_{jil} \nabla_{m} v. \end{aligned} $$

It follows from (2.10)

2.11 $$ \nabla_{ii} G \geq\nabla_{11ii} u + 2 s \bigl( \vert \nabla_{i1} u \vert ^{2} + \nabla _{1} u \nabla_{1ii} u\bigr) - C ( 1 + G), $$

and

2.12 $$ \begin{aligned}[b] \nabla_{ii} G + \gamma \Delta G \geq{}& \nabla_{11ii} u + 2 s \bigl( \vert \nabla_{i1} u \vert ^{2} + \nabla_{1} u \nabla_{1ii} u\bigr) + \gamma\nabla _{11} (\Delta u) \\ & {} + 2 s \gamma \bigl( \vert \nabla_{i1} u \vert ^{2} + \nabla_{1} u \nabla_{1} (\Delta u) \bigr) - C (1 + G). \end{aligned} $$

Differentiating equation (1.16) once at $x_{0}$, we obtain for $1 \leq k \leq n$,

2.13 $$ \nabla_{k} F = F^{ii} \nabla_{k} T_{ii} = \psi_{x_{k}} + \psi_{z} \nabla_{k} u + \nabla_{k} \beta_{\varepsilon}(u - h). $$

It is easy to see that

2.14 $$ \begin{aligned}[b] F^{ii} \nabla_{1} (\nabla_{ii} u + \gamma\Delta u) &= F^{ii} \nabla_{1} \biggl(T_{ii} [u] - s \vert \nabla_{i} u \vert ^{2} + \frac{t}{2} \vert \nabla u \vert ^{2} - \chi_{ii} \biggr) \\ &\geq\nabla_{1} F - 2s F^{ii} \nabla_{i} u \nabla_{1i} u + t \nabla_{k} u \nabla_{1k} u\sum _{i} F^{ii} - \sum _{i} F^{ii} \end{aligned} $$

and that

2.15 $$ \begin{aligned}[b] F^{ii} \nabla_{11} (\nabla_{ii} u + \gamma\Delta u) = {}& F^{ii} \nabla_{11} \biggl(T_{ii} [u] - s \vert \nabla_{i} u \vert ^{2} + \frac {t}{2} \vert \nabla u \vert ^{2} - \chi_{ii} \biggr) \\ \geq{}& F^{ii} \nabla_{11} T_{ii} [u] - 2s F^{ii} \bigl( \nabla_{i} u \nabla_{11i} u + \vert \nabla_{1i} u \vert ^{2} \bigr) \\ &{} + t \sum_{k} \bigl(\nabla_{k} u \nabla_{11k} u + \vert \nabla_{1k} u \vert ^{2} \bigr) \sum_{i} F^{ii} - C \sum _{i} F^{ii}. \end{aligned} $$

With (2.9) we see

2.16 $$ \begin{aligned}[b] 2s \nabla_{i} u \nabla_{11i} u & \leq2s \nabla_{i} u (\nabla_{i} G - 2s \nabla_{1} u \nabla_{i1} u) + C \\ & \leq- 4s^{2} \nabla_{i} u \nabla_{1} u \nabla_{i1} u + C \bigl( 1 + G \vert \nabla\phi \vert \bigr), \end{aligned} $$

and similarly

2.17 $$ t \nabla_{k} u \nabla_{11k} u \geq- 2st \nabla_{k} u \nabla_{1} u \nabla _{k1} u - C \bigl( 1 + G \vert \nabla\phi \vert \bigr). $$

With (2.12), (2.14)–(2.17), and the concavity of *F*, we derive

2.18 $$ \begin{aligned} F^{ii} ( \nabla_{ii} G + \gamma\Delta G) \geq{}& \nabla_{11} F + 2s \nabla_{1} u \nabla_{1} F + (2s \gamma+ t) \sum _{k} \vert \nabla_{1k} u \vert ^{2} \sum F^{ii} \\ & {} - C \biggl(G + G \vert \nabla\phi \vert + \sum _{j,k} \vert \nabla_{jk} u \vert \biggr) \\ \geq{} & \nabla_{11} F + 2s \nabla_{1} u \nabla_{1} F - C \bigl(G^{2} + G \vert \nabla\phi \vert \bigr). \end{aligned} $$

By (1.9) and $\beta_{\varepsilon}'' > 0$ it follows from (2.6) and (2.18) that

2.19 $$ \begin{aligned}[b] &F^{ii} \bigl( \nabla_{ii} \phi- \vert \nabla_{i} \phi \vert ^{2} \bigr) + \gamma \bigl( \Delta\phi- \vert \nabla\phi \vert ^{2} \bigr) \sum F^{ii} \\ &\quad\leq C \bigl(G + \vert \nabla\phi \vert \bigr) \sum F^{ii} + \biggl(\frac {C}{G} - 1 \biggr)\beta'_{\varepsilon} (u - h). \end{aligned} $$

Let

$$\phi:= \eta(w) = \biggl(1 - \frac{w}{2 a} \biggr)^{-1/2} , \quad w = \frac{ \vert \nabla u \vert ^{2}}{2}, $$

where $a > \sup_{M} w$ is a constant to be determined. We have

$$1 \leq\eta< \sqrt{2} , \quad\eta' = \frac{\eta^{3}}{4 a}, \qquad \eta'' = \frac{3 \eta^{\prime2}}{\eta} $$

and

2.20 $$ \nabla_{ii} \phi- \vert \nabla_{i} \phi \vert ^{2} = \eta' \nabla_{ii} w + \bigl( \eta'' - \eta^{\prime2} \bigr) \vert \nabla_{i} w \vert ^{2} \geq\eta' \nabla_{ii} w. $$

Next, by (2.14)

2.21 $$ \begin{aligned}[b] &F^{ii} ( \nabla_{ii} w + \gamma\Delta w ) \\ &\quad= F^{ii} \biggl(\sum_{l} \vert \nabla_{il} u \vert ^{2} + \gamma\sum _{k,l} \vert \nabla_{kl} u \vert ^{2} \biggr) + F^{ii} \nabla_{l} u \bigl( \nabla_{iil} u + \gamma\Delta(\nabla_{l} u) \bigr) \\ &\quad\geq F^{ii} \nabla_{l} u \biggl(\nabla_{lii} u + \gamma\sum_{k}\nabla_{lkk} u \biggr) + \bigl(\gamma G^{2} - C G \bigr) \sum F^{ii} \\ &\quad\geq- C \beta'_{\varepsilon} (u - h) + \bigl(\gamma G^{2} - C G \bigr) \sum F^{ii}. \end{aligned} $$

Combining (2.19), (2.20), (2.21), and $|\nabla \phi| \leq C \eta' G$, we have

2.22 $$ \eta' \bigl(\gamma G^{2} - C G \bigr) \sum F^{ii} \leq C \bigl(G + \eta' G \bigr) \sum F^{ii} + \biggl(\frac{C}{G} - 1 + C \eta' \biggr)\beta '_{\varepsilon} (u - h). $$

We could assume that $G \geq2 C$. When $a > 2 C$, the coefficient of $\beta'_{\varepsilon}(u - h)$ is negative. Then we can derive $G \leq\frac{4 a C}{\gamma}$. □

To derive the boundary estimates for $\nabla^{2} u$, we note that $\operatorname{tr} (s du \otimes du - \frac{t}{2} |\nabla u|^{2} g + \chi ) \leq C$ on *M̄*, where *C* is independent of *ε*, though it may depend on $|u|_{C^{1}(\bar{M})}$. As in [1, 4], let *H* be the solution to

$$\textstyle\begin{cases} (1 + n \gamma )\Delta H + C = 0 & \mbox{in } M, \\ H = \varphi& \mbox{on } \partial M. \end{cases} $$

Then we have $u \leq H$ in *M* by the maximum principle and $\beta_{\varepsilon}(u - h) \equiv0$ in $M_{\delta}= \{x \in M: \operatorname{dist} (x, \partial M) \leq\delta\}$, where *δ* is sufficiently small. Thus,

2.23 $$ \textstyle\begin{cases} f (\lambda( g^{-1} T[u] )) = \psi[u] & \mbox{in } M_{\delta},\\ u = \varphi& \mbox{on } \partial M. \end{cases} $$

By the same arguments of Sect. 4 in [8], we obtain that

2.24 $$ \sup_{\partial M} \bigl\vert \nabla^{2} u \bigr\vert \leq C, $$

where *C* depends on $|u|_{C^{1}(\bar{M})}$ and other known data.

Combining (2.1) and (2.24), we therefore get the full estimates for second order derivatives.

## Gradient estimates, maximum principle, and existence

For the gradient estimates, we have the following theorem.

### Theorem 3.1

*Assume that* (1.5)*–*(1.8) *hold*. *Let* $u \in C^{3} (\bar{M})$ *be an admissible solution to* (1.16). *Then*

3.1 $$ \sup_{M} \vert \nabla u \vert \leq C \Bigl(1 + \sup_{\partial M} \vert \nabla u \vert \Bigr), $$

*where* *C* *depends on* $|u|_{C^{0} (\bar{M})}$ *and other known data*.

### Proof

Suppose that $we^{\phi}$, where $w = \frac{|\nabla u|^{2}}{2}$ and $\phi = \phi(u)$ to be determined satisfying that $\phi' (u) > 0$, achieves a maximum at an interior point $x_{0} \in M$. As before, we choose a smooth orthonormal local frame $e_{1}, \ldots, e_{n}$ about $x_{0}$ such that $\nabla_{e_{i}} e_{j} = 0$ at $x_{0}$ and $\{T_{ij} (x_{0})\}$ is diagonal. Differentiating $we^{\phi}$ at $x_{0}$ twice, we have

3.2 $$ \nabla_{i} w + w \nabla_{i} \phi= 0 $$

and

3.3 $$ \nabla_{ii} w - w (\nabla_{i} \phi)^{2} + w \nabla_{ii} \phi\leq0. $$

Differentiating *w*, we see

$$\nabla_{i} w = \sum_{k} \nabla_{k} u \nabla_{ik} u, \qquad \nabla_{ii} w = \sum_{k} (\nabla_{ik} u)^{2} + \sum_{k} \nabla_{k} u \nabla_{iik} u . $$

Using (3.2) it follows from (3.3) that

3.4 $$ F^{ii} \biggl(\delta_{kl} - \frac{\nabla_{k} u \nabla_{l} u}{2 w} \biggr) \nabla_{ik} u \nabla_{il} u + F^{ii} \nabla_{k} u \nabla_{iik} u - w F^{ii} \biggl( \frac{(\nabla_{i} \phi)^{2}}{2} - \nabla_{ii} \phi \biggr) \leq0 $$

and

3.5 $$ \sum_{i,k,l} \biggl( \delta_{kl} - \frac{\nabla_{k} u \nabla_{l} u}{2 w} \biggr) \nabla_{ik} u \nabla_{il} u + \nabla_{k} u \Delta(\nabla_{k} u) - \frac{w}{2} \vert \nabla\phi \vert ^{2} + w \Delta\phi \leq0. $$

Note that the first term in (3.4) and (3.5) is nonnegative. Multiply $\gamma\sum F^{ii}$ to (3.5) and add what we got to (3.4). Thus, by (2.9) we obtain

3.6 $$ \begin{aligned}[b] & F^{ii} \nabla_{k} u (\nabla_{kii} u + \gamma\nabla_{k} \Delta u ) - \frac{w}{2} F^{ii} \bigl( \vert \nabla_{i} \phi \vert ^{2} + \gamma \vert \nabla\phi \vert ^{2} \bigr) \\ &\quad{}+ w F^{ii} ( \nabla_{ii} \phi+ \gamma\Delta\phi ) \leq C \vert \nabla u \vert ^{2} \sum F^{ii} . \end{aligned} $$

Now we compute the first term in (3.6). Firstly, we have

$$\nabla_{i} \phi= \phi' \nabla_{i} u, \qquad \nabla_{ii} \phi= \phi' \nabla_{ii} u + \phi'' (\nabla_{i} u)^{2}. $$

Using (3.2), we easily get that

3.7 $$ \begin{aligned}[b] & F^{ii} \nabla_{k} u (\nabla_{kii} u + \gamma\nabla_{k} \Delta u ) \\ & \quad= F^{ii} \nabla_{k} u \nabla_{k} \biggl( T_{ii} - s \vert \nabla_{i} u \vert ^{2} + \frac{t}{2} \vert \nabla u \vert ^{2} - \chi_{ii} \biggr) \\ & \quad= \nabla_{k} u \nabla_{k} (\psi+ \beta_{\varepsilon}) + w \phi ' F^{ii} \bigl( 2 s \vert \nabla_{i} u \vert ^{2} - t \vert \nabla u \vert ^{2} \bigr) - F^{ii} \nabla_{k} u \nabla_{k} \chi_{ii}. \end{aligned} $$

By the homogeneity of *F*, we also get

3.8 $$ \begin{aligned}[b] & F^{ii} ( \nabla_{ii} \phi+ \gamma\Delta\phi ) \\ & \quad= \phi'' F^{ii} \bigl( \vert \nabla_{i} u \vert ^{2} + \gamma \vert \nabla u \vert ^{2} \bigr) + \phi' F^{ii} \biggl( T_{ii} - s \vert \nabla_{i} u \vert ^{2} + \frac {t}{2} \vert \nabla u \vert ^{2} - \chi_{ii} \biggr) \\ & \quad= \phi'' F^{ii} \bigl( \vert \nabla_{i} u \vert ^{2} + \gamma \vert \nabla u \vert ^{2} \bigr) + \phi' \biggl(F - s F^{ii} \vert \nabla_{i} u \vert ^{2} + \frac{t}{2} F^{ii} \vert \nabla u \vert ^{2} - F^{ii} \chi_{ii} \biggr). \end{aligned} $$

According to (3.7) and (3.8), it follows from (3.6)

3.9 $$ \begin{aligned}[b] & \gamma \vert \nabla u \vert ^{2} \biggl( \phi'' - \frac{1}{2} \bigl(\phi'\bigr)^{2} - \frac {t}{2 \gamma} \phi' \biggr) \sum F^{ii} + \biggl( \phi'' - \frac{1}{2} \bigl(\phi' \bigr)^{2} + s \phi' \biggr) F^{ii} ( \nabla_{i} u)^{2} \\ & \quad\leq- \phi' \bigl(\psi+ \beta_{\varepsilon} - F^{ii} \chi_{ii} \bigr) + C \sum F^{ii} - \frac{\nabla_{k} u \nabla_{k} (\psi+ \beta_{\varepsilon})}{w} \\ & \quad\leq- \biggl( \phi' \beta_{\varepsilon} + \frac{\nabla_{k} u \nabla_{k} (u - h) \beta'_{\varepsilon}}{w} \biggr) + C \sum F^{ii} + C . \end{aligned} $$

Let

$$\phi(u) = v^{- a}, \qquad v = 1 - u + \sup_{M} u. $$

We have

$$\phi' (u) = a v^{- a - 1}, \qquad\phi'' (u) = \frac{(a + 1 )\phi '}{v} , $$

and

$$\phi'' - \frac{1}{2} \bigl( \phi'\bigr)^{2} = \phi' \biggl( \frac{a + 1}{v} - \frac{a v^{- a}}{2v} \biggr) \geq\frac{\phi' a}{2v} > 0 $$

since $v^{- a} \leq1$. When $|\nabla u (x_{0})|$ is sufficiently large, we see $\nabla_{k} u \nabla_{k} (u - h) > 0$. Hence we have that the first term on the right-hand side of (3.9) is negative as $\beta_{\varepsilon}, \beta'_{\varepsilon} > 0$. From (3.9) and (1.9) when *a* is sufficiently large, we then obtain that

3.10 $$ \frac{\phi' a \gamma \vert \nabla u \vert ^{2} }{4 v} \leq C, $$

from which we conclude that (3.1) holds. □

In order to prove (1.19), it remains to bound $\sup_{M} |u| + \sup_{\partial M} |\nabla u|$. We quote two lemmas in [8], the ingredients of whose proofs are the maximum principle.

### Lemma 3.2

*If either* (1.10) *or* (1.11) *holds*, *then any admissible solution* *u* *of* (1.16) *admits the a priori bound*

3.11 $$ \sup_{M} u \leq c_{0}. $$

### Lemma 3.3

*If* *u* *is admissible such that* $\operatorname{tr} T[u] \geq0$ *and* $|u|_{C^{0}(M)} \leq\mu$, *then*

3.12 $$ \sup_{\partial M} \nabla_{\nu}u \leq c_{1} (\mu), $$

*where* *ν* *is the interior unit normal to* *∂M*.

Now with the above two lemmas and the fact $\nabla_{\nu}u \geq\nabla _{\nu}\underline{u}$ on *∂M* when $u \in\mathscr{U}$, we then have the following.

### Theorem 3.4

*Suppose that* (1.5)*–*(1.8), *and either* (1.10) *or* (1.11) *hold*. *Then*, *for* $u \in\mathscr{U}$, (1.19) *holds*.

Therefore, the uniform estimates (1.19) ensure that there exist a subsequence $\{u_{\varepsilon_{k}}\}$ of $\{u_{\varepsilon}\}$ and a function $u \in C^{1,1} (\bar{M})$ such that $u_{\varepsilon_{k}} \rightarrow u$ in *M* as $\varepsilon_{k} \rightarrow0$. It is easy to verify that *u* satisfies (1.3) and (1.4) and $u \in C^{3, \alpha} (E)$ for any $\alpha\in(0, 1)$. Consequently, Theorem 1.1 is established.

## References

[1] Bao G.-J., Dong W.-S., Jiao H.-M. (2015). Regularity for an obstacle problem of Hessian equations on Riemannian manifolds. J. Differ. Equ..

[2] Caffarelli L.A., Nirenberg L., Spruck J. (1985). The Dirichlet problem for nonlinear second order elliptic equations III, functions of the eigenvalues of the Hessian. Acta Math..

[3] Crandall M., Ishii H., Lions P. (1992). User’s guide to viscosity solutions of second order partial differential equations. Bull. Am. Math. Soc..

[4] Dong W.-S., Wang T.-T., Bao G.-J. (2016). A priori estimates for the obstacle problem of Hessian type equations on Riemannian manifolds. Commun. Pure Appl. Anal..

[5] Evans L.C. (1982). Classical solutions of fully nonlinear, convex, second order elliptic equations. Commun. Pure Appl. Math..

[6] Gerhardt C. (1973). Hypersurfaces of prescribed mean curvature over obstacles. Math. Z..

[7] Guan B. (1999). The Dirichlet problem for Hessian equations on Riemannian manifolds. Calc. Var. Partial Differ. Equ..

[8] Guan B. (2008). Complete conformal metrics of negative Ricci curvature on compact manifolds with boundary. Int. Math. Res. Not..

[9] Guan B. (2014). Second order estimates and regularity for fully nonlinear elliptic equations on Riemannian manifolds. Duke Math. J..

[10] Guan, B.: The Dirichlet problem for fully nonlinear elliptic equations on Riemannian manifolds. arXiv:1403.2133

[11] Guan B., Jiao H.-M. (2015). Second order estimates for Hessian type fully nonlinear elliptic equations on Riemannian manifolds. Calc. Var. Partial Differ. Equ..

[12] Guan B., Jiao H.-M. (2016). The Dirichlet problem for Hessian type fully nonlinear elliptic equations on Riemannian manifolds. Discrete Contin. Dyn. Syst..

[13] Guan P.-F., Wang G.-F. (2003). Local estimates for a class of fully nonlinear equations arising from conformal geometry. Int. Math. Res. Not..

[14] Jiao H.-M. (2016). $C^{1,1}$ regularity for an obstacle problem of Hessian equations on Riemannian manifolds. Proc. Am. Math. Soc..

[15] Jiao H.-M., Wang Y. (2014). The obstacle problem for Hessian equations on Riemannian manifolds. Nonlinear Anal. TMA.

[16] Krylov N.V. (1983). Boundedly inhomogeneous elliptic and parabolic equations in a domain. Izvestia Math. Ser..

[17] Lee K. (2001). The obstacle problem for Monge–Ampère equation. Commun. Partial Differ. Equ..

[18] Liu J.-K., Zhou B. (2013). An obstacle problem for a class of Monge–Ampère type functionals. J. Differ. Equ..

[19] Oberman A. (2007). The convex envelope is the solution of a nonlinear obstacle problem. Proc. Am. Math. Soc..

[20] Oberman A., Silvestre L. (2011). The Dirichlet problem for the convex envelope. Trans. Am. Math. Soc..

[21] Savin O. (2005). The obstacle problem for Monge–Ampere equation. Calc. Var. Partial Differ. Equ..

[22] Schoen R. (1984). Conformal deformation of a Riemannian metric to constant scalar curvature. J. Differ. Geom..

[23] Trudinger N.S. (1995). On the Dirichlet problem for Hessian equations. Acta Math..

[24] Urbas J. (2002). Hessian equations on compact Riemannian manifolds. Nonlinear Problems in Mathematical Physics and Related Topics, II.

[25] Viaclovsky J.A. (2000). Conformal geometry, contact geometry, and the calculus of variations. Duke Math. J..

[26] Viaclovsky J.A. (2006). Conformal Geometry and Fully Nonlinear Equations.

[27] Xiong J.-G., Bao J.-G. (2011). The obstacle problem for Monge–Ampère type equations in non-convex domains. Commun. Pure Appl. Anal..

